# Noggin Combined With Human Dental Pulp Stem Cells to Promote Skeletal Muscle Regeneration

**DOI:** 10.1155/sci/2812390

**Published:** 2024-12-28

**Authors:** Meng-Han Zhang, Li-Ming Yu, Wei-Hua Zhang, Jia-Jia Deng, Bing-Jing Sun, Mei-Hua Chen, Wei Huang, Jiao Li, Hua He, Xin-Xin Han, Yue-Hua Liu

**Affiliations:** ^1^Shanghai Key Laboratory of Craniomaxillofacial Development and Diseases, Shanghai Stomatological Hospital and School of Stomatology, Fudan University, Shanghai, China; ^2^School of Stomatology Affiliated to Medical College, Zhejiang University, Hangzhou, China; ^3^Department of Neurosurgery, Third Affiliated Hospital of Second Military Medical University, Shanghai, China

## Abstract

A proper source of stem cells is key to muscle injury repair. Dental pulp stem cells (DPSCs) are an ideal source for the treatment of muscle injuries due to their high proliferative and differentiation capacities. However, the current myogenic induction efficiency of human DPSCs hinders their use in muscle regeneration due to the unknown induction mechanism. In this study, we treated human DPSCs with Noggin, a secreted antagonist of bone morphogenetic protein (BMP), and discovered that Noggin can effectively promote myotube formation. We also found that Noggin can accelerate the skeletal myogenic differentiation (MyoD) of DPSCs and promote the generation of Pax7^+^ satellite-like cells. Noggin increased the expression of myogenic markers and the transcriptional and translational abundance of satellite cell (SC) markers in DPSCs. Moreover, BMP4 inhibited Pax7 expression and activated p-Smad1/5/9, while Noggin eliminated BMP4-induced p-Smad1/5/9 in DPSCs. This finding suggests that Noggin antagonizes BMP by downregulating p-Smad and facilitates the MyoD of DPSCs. Then, we implanted Noggin-pretreated DPSCs combined with Matrigel into the mouse tibialis anterior muscle with volumetric muscle loss (VML) and observed a 73% reduction in the size of the defect and a 69% decrease in scar tissue. Noggin-treated DPSCs can benefit the Pax7^+^ SC pool and promote muscle regeneration. This work reveals that Noggin can enhance the production of satellite-like cells from the MyoD of DPSCs by regulating BMP/Smad signaling, and these satellite-like cell bioconstructs might possess a relatively fast capacity for muscle regeneration.

## 1. Introduction

Muscle stem cells, also called satellite cells (SCs) and defined by the transcription factor paired box 7 (Pax7), are responsible for skeletal muscle maintenance and repair. Once activated after injury, SCs enter the cell cycle, commit to myogenic progenitor cells (MPCs), differentiate into myoblast cells, and finally fuse into multinucleated myotubes. A subset of SCs undergoes asymmetric division, renewing the SC pool [1]. However, endogenous SCs may be inefficient in responding to severe trauma or chronic degenerative diseases such as malnutrition, neuromuscular diseases, and sleep apnea [2–4]. When more than 20% of the muscle volume is missing, as in volumetric muscle loss (VML), endogenous self-repair is hindered [5]. Moreover, SCs are found in very limited numbers in adult muscles and exhibit undesirable amplification activity in vitro [6]; thus, these SCs are not the appropriate stem cell pool for muscle repair.

Dental pulp stem cells (DPSCs) are a type of mesenchymal stem cells (MSCs) that exhibit therapeutic potential for tissue regeneration [7]. These cells are easily established from dental pulp [8] and have several advantages compared to other types of MSCs: better proliferative potential and migratory capabilities [9], a simpler primary isolation method, a higher success rate in long-term in vitro culture [10], and long-term storage without losing their stemness and ability to differentiate [11, 12]. Kerkis et al. [13] found the therapeutic effect of human DPSCs on Golden Retrievers with muscular dystrophy. Recently, DPSCs were shown to undergo myogenic differentiation (MyoD), and these myogenic lineage cells could treat the dystrophic muscles of mdx/SCID mice [14]. However, the current myogenic efficiency is still quite low [15]. Here, we searched for novel factors to develop appropriate MyoD systems for DPSCs.

MyoD is regulated by genes such as MyoD1, myogenic factor 5 (Myf5), myogenic regulatory factor 4 (MRF4), Desmin, and myosin heavy chain (MyHC) to determine cell fate, including myoblast differentiation into myocytes, fusion into myotubes, and maintenance of myofibers [15, 16]. Factors, molecules, or stimuli can be applied to affect MSCs' fate and commitment [17], and biocompatible scaffolds are provided for the natural environment in the use of MSCs [12]. A recent RNA-seq analysis identified that repressed bone morphogenetic protein (BMP) and activated Wnt signaling pathways are required for nascent somites during embryonic myogenesis [18]. Wnt activation alone can induce pluripotent stem cells (PSCs) committed to myogenic progenitors. It further requires BMP inhibition to maintain progenitor fate, followed by the myogenic program to generate myofibers and associated Pax7^+^ cells [19]. These results suggest that Wnt activation or BMP antagonism might be important mechanisms in myogenic regulation.

Noggin, a secreted homodimeric glycoprotein, is promoted by Wnt-1 in medial somites and is required for embryonic somite and skeletal patterning [20, 21]. Then, Noggin was found to inhibit the actions of BMP-2, -4, -5, -7, -13, and -14, thus blocking Smad-dependent signaling [22]. BMP signaling has been shown to play a role in controlling SC lineage progression during embryonic myogenesis [23], and these SC progeny secrete BMP antagonists, such as Noggin, for the differentiation of muscle progenitor cells [24]. Noggin-null mice display defective skeletal muscle fibers [25]. However, the effect of Noggin on the MyoD of DPSCs has not yet been reported.

In the present study, Noggin was applied to MyoD systems for DPSCs. Then, we implanted these pretreated DPSCs combined with Matrigel as scaffolds into the mouse tibialis anterior muscle with VML. Here, we explore the DPSCs repair capacity in muscle injury.

## 2. Materials and Methods

### 2.1. Cell Culture and Identification

**Figure 1:**
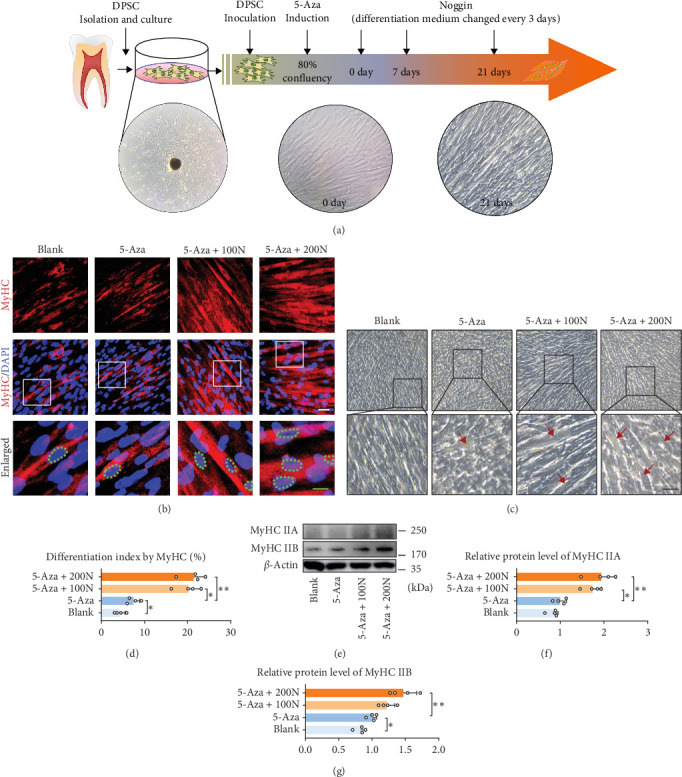
Noggin promotes the formation of myotube in DPSCs. (a) In vitro myogenic differential system for DPSCs. (b) Formation of myotubes and cell nuclei was assessed using immunofluorescence staining with MyHC (red) and DAPI (blue) on Day 21, respectively. Green dotted circle indicates MyHC-positive merged myonuclei; white scale bar, 10 µm; green scale bar, 5 µm. (c) Cell morphology of DPSCs induced by 5-Aza with or without Noggin (100 ng/mL or 200 ng/mL) on Day 21. Red arrows indicates Myotube-like cells; Scale bar, 50 µm. (d) The differentiation index is presented as the ratio of MyHC-positive nuclei to total nuclei (*n* = 4). (e) Protein expression of MyHC IIA and MyHC IIB was assessed using western blotting on Day 21. (f, g) Semiquantitative analysis of the expression ratio of (f) MyHC IIA/*β*-actin and (g) MyHC IIB/*β*-actin (*n* = 4). The difference between blank and 5-Aza or between 5-Aza, 5-Aza + 100N and 5-Aza + 200N were presented as *⁣*^*∗*^*p* < 0.05 and *⁣*^*∗∗*^*p* < 0.01. 100N = 100 ng/mL Noggin; 200N = 200 ng/mL Noggin. DAPI, 4′,6-diamidino-2-phenylindole; DPSCs, dental pulp stem cells; MyHC, myosin heavy chain.

DPSCs were acquired as previously described (Figure 1a,b, Table S1) [26] and cultured in *α*-MEM (Gibco, NY, USA) with 10% fetal bovine serum (FBS, Gibco, NY, USA) and 1% penicillin-streptomycin (Gibco, NY, USA) at 37°C in the presence of 5% CO_2_ and 95% air. Flow cytometric analysis was used to determine the cell surface markers present on DPSCs. DPSCs were identified with phycoerythrin (PE)-conjugated antibodies against human CD29 (#555443) and FITC-conjugated antibodies against human CD90 (#555595) from BD Biosciences (CA, USA), fluorescein isothiocyanate (FITC)-conjugated antibodies against human CD44 (#11-0441-85) from eBioscience (CA, USA), and PE-conjugated antibodies against human CD34 (#343605) and FITC-conjugated antibodies against human CD45 (#304005) from BioLegend (CA, USA) and analyzed using FlowJo software (FlowJo, OR, USA). Cell cycle analysis was also performed after staining for 24 h with propidium iodide (PI) (Beyotime, Shanghai, China) according to the manufacturer's protocol using flow cytometry (ACEA NovoCyte, CA, USA).

### 2.2. MyoD Assay

DPSCs were seeded on 6-well plates at a cell density of 4000 cells/cm^2^ in an expansion medium consisting of *α*-MEM with 10% FBS for adherence. When the confluence reached 80%, the culture medium was replaced first with a myogenic induction medium containing the following for 24 h: IMDM (Gibco, NY, USA) + 2% FBS + 1 μM 5-Aza-2′-deoxycytidine (5-Aza) (an important trigger for myogenic commitment differentiation [14]) (Sigma-Aldrich, CA, USA) [27]. IMDM + 2% FBS without 5-Aza served as the base control medium. Subsequently, cells were rinsed three times in phosphate-buffered saline (PBS, HyClone, UT, USA) and then transferred to the following differentiation medium: IMDM with 10% FBS (defined as Day 0) (Figure 1a). The differentiation medium was supplemented with or without Noggin (100 ng/mL = 100N or 200 ng/mL = 200N) or 50 ng/mL BMP4. Noggin (#10267) from Sino Biological (Beijing, China) and BMP4 (HZ-1045) from HumanZyme (IL, USA) were used. Cells were cultured for 3 weeks, the differentiation medium was changed every 3 days, and the cells were observed under a microscope to confirm the formation of myotubes.

### 2.3. VML Defect Modeling and Stem Cell Transplantation

Animal protocols were approved by Cyagen IACUC (No. ACU20-A034). Male Balb/C mice (8 weeks old) were purchased from Shanghai Bikai Biotechnology. All animals underwent surgery to create a muscle defect by unilaterally resecting the tibialis anterior muscle under 2% pentobarbital sodium anesthesia. The thin layer of fascia covering the tibialis anterior muscle was dissected away, and a 2 × 5 mm area was stained with hematoxylin. After a blunt separation, a 2 mm-deep cut was made around the stained hematine, creating a 2 × 5 × 2 mm defect. This VML defect accounted for a loss of ~40% of the tibialis anterior muscle. DPSCs were cultured and differentiated by 5-aza and Noggin treatment, and Matrigel (Corning, NY, USA) was used to provide a scaffold for stem cell transplantation. Matrigel alone (*n* = 6) or Matrigel combined with Noggin-treated (*n* = 6) or untreated DPSCs (*n* = 6) was used to fill the dissected area. If no immediate transplantation was performed (*n* = 6), the incision was sutured closed. Mice were allowed to heal for 30 days.

### 2.4. Hematoxylin and Eosin Staining

The tibialis anterior muscles were excised from mice in the four groups and fixed in ice-cold 4% PFA. After dehydration in a graded alcohol series, the tissue was embedded in paraffin and cut into 5 µm-thick sections by using a Leica RM2255 rotary microtome (Leica Microsystems, Mannheim, Germany). After deparaffinization in xylene and hydration through a graded alcohol series to ddH_2_O, hematoxylin, and eosin (Servicebio, Wuhan, China), staining was performed according to the manufacturer's protocols.

### 2.5. RNA Extraction and Real-Time Polymerase Chain Reaction (PCR)

Total RNA was extracted from the cells using TRIzol (Life Technologies, CA, USA). The RNA concentration was quantified using a spectrophotometer by measuring the OD260/280 ratio (1.80–1.95). DNA was removed by gDNase at 42°C for 3 min. cDNA was reverse transcribed using a FastQuant RT Kit (Tiangen, Beijing, China) at 42°C for 15 min and at 95°C for 3 min. Subsequently, SYBR Green Premix (Tiangen, Beijing, China) was used for the amplification of cDNA on a Light Cycler 96 system (Roche, Basel, Switzerland) with each primer at 0.6 mM and 150 ng of cDNA template. The thermocycling conditions were as follows: 95°C for 15 min, followed by 40 cycles at 95°C for 10 s and at 60°C for 32 s. Transcript levels were normalized using *β*-actin as a housekeeping gene and analyzed with the 2^−∆∆Ct^ method. The primer sequences can be found in Table S2.

### 2.6. Western Blot Analysis

Total protein was extracted in RIPA lysis buffer (Thermo Fisher Scientific, MA, USA) plus protease/phosphatase inhibitor cocktail (#5872, CST, MA, USA). Lysate proteins (25 mg) were loaded onto SDS-polyacrylamide gels (7.5% or 10%) and subsequently transferred onto PVDF membranes. Target proteins on the membranes were incubated overnight at 4°C with primary antibodies diluted 1:1000–1:200 in 1 × TBS-Tween (TBS: 0.05 M Tris, 0.15 M NaCl [pH 7.5]; with 0.2% Tween-20). Antibodies against *β*-actin (abs830031; Absin Bioscience, Shanghai, China), Pax7 (ab187339), and Myf5 (ab125078) from Abcam; MyHC IIB (BF-F3) and MyHC IIA (SC-71) from Developmental Studies Hybridoma Bank (DSHB, ID, USA); p-Smad 1/5/9 (#13820) and Smad 1 (#6944) from Cell Signaling Technology (CST, MA, USA); and MyoD (#18943), Desmin (#60226), MRF4 (#11754), Pax3 (#21383), Six1 (#10709), Eya2 (#11314), ID1 (#18475), and MSX1 (#17678) from Proteintech (IL, USA) were used. After intensive washing, the membranes were incubated with HRP-conjugated secondary antibodies (#7076, anti-mouse IgG; #7074, antirabbit IgG, both from CST) for 1–2 h. The membranes were visualized using Super Signal West Dura substrate (Thermo Scientific, MA, USA), and the bands were detected with an AI600 imager (GE Healthcare, IL, USA).

### 2.7. Immunofluorescence Microscopy

Cells, myofibers, and sections were fixed with 4% paraformaldehyde and placed in PBS containing 0.25% Triton X-100 for 10 min. After the samples were blocked with 3% bovine serum albumin (BSA, Solarbio, Beijing, China) in PBS for 30 min at room temperature, they were then incubated with the primary antibody in PBS containing 2.5% BSA at 4°C overnight. In addition to the primary antibodies listed above, antibodies against Pax7 (PAX7, DSHB), CD34 (#14486, Proteintech), human Lamin A/C (MBS477941, MyBioSource), human nucleoli (hNu) (ab190710, Abcam), and Laminin (ab11575, Abcam) were used. After washing, the samples were incubated with anti-mouse Alexa-594 or Alexa-488, or antirabbit Alexa-594 or Alexa-488 (Jackson ImmunoResearch, PA, USA), and then with 4′,6-diamidino-2-phenylindole (DAPI). Images of the cells were captured using a microscope (Leica Microsystems, Mannheim, Germany) and analyzed with ImageJ.

### 2.8. Cell Cycle Analysis

The cells were harvested and fixed in 70% precooled ethanol overnight at 4°C. After washing with PBS the next day, the cells were incubated with a solution containing RNase A and 50 µg/mL of PI (C1052, Beyotime, Shanghai, China) for 30 min at 37°C. Then, the cell cycle analysis was examined using a Novocyte 2040R flow cytometer (ACEA Biosciences, Hangzhou, China) with a cell count of ~0.5 × 10^4^ ~ 2.0 × 10^4^ frequency of G1, S, and G2 phases using NovoExpress software version 1.4.1 (ACEA Novocyte). The results were graphed using GraphPad Prism software.

### 2.9. Statistical Analysis

We performed statistical analyses using GraphPad Prism 5.0. Data are expressed as the mean ± standard deviation. The protein quantities were analyzed using semi-quantitative analysis. Comparisons between groups were performed using the Mann‒Whitney *U* test or Kruskal‒Wallis *H* test with multiple comparisons. Statistical significance was defined as *⁣*^*∗*^*p* < 0.05, *⁣*^*∗∗*^*p* < 0.01, *⁣*^*∗∗∗*^*p* < 0.001.

## 3. Results

### 3.1. Noggin Promotes the Formation of Myotubes in DPSCs

We cultured and identified DPSCs (Figure S1) and found that 5-Aza can induce MyoD in DPSCs by increasing myogenic markers (Figure S2) but with low efficiency. To examine whether Noggin could affect the MyoD of DPSCs, we treated DPSCs with Noggin after 5-Aza induction (Figure 1a). First, we found that Noggin had no significant effect on the proliferation of DPSCs (Figure S3 a–c) but increased myotube formation in DPSCs (Figure 1b,c). A more pronounced myotube morphology was observed after treatment with the Noggin protein for 21 days, and the differential index of multinuclear myotubes increased to ~20% (Figure 1d). The protein expression levels of MyHC (Figure 1e), such as MyHCIIA (Figure 1f) and MyHCIIB (Figure 1g), were significantly increased compared to those in the control group. This finding implies that Noggin might facilitate the formation of myotubes in DPSCs.

### 3.2. Noggin Accelerates the Progression of MyoD in DPSCs

**Figure 2:**
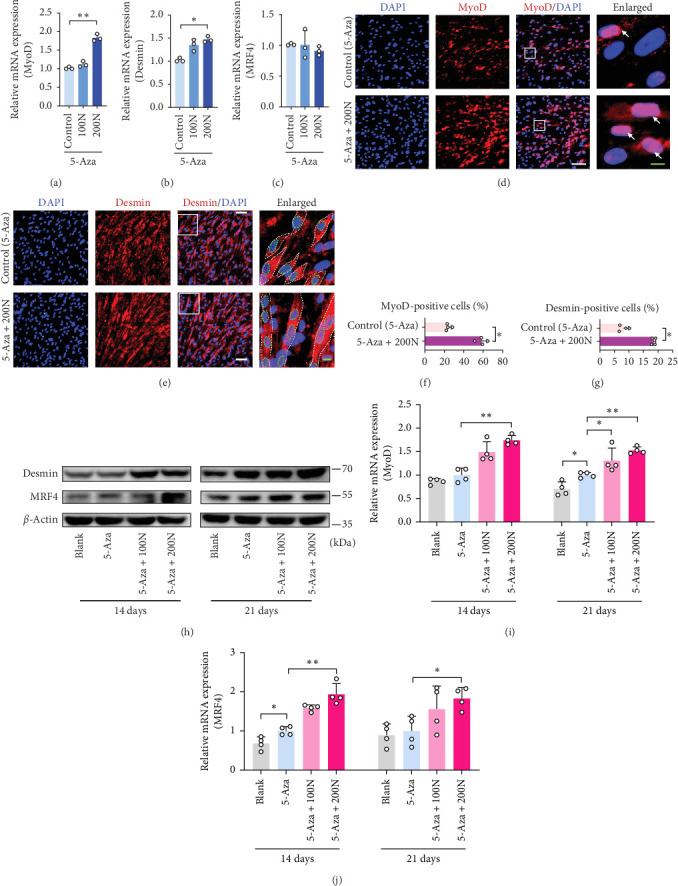
Noggin accelerates the progress of myogenic differentiation of DPSCs. (a–c) The levels of relative mRNAs, including (a) MyoD, (b) Desmin, and (c) MRF4, were assessed using quantitative PCR (*n* = 3). (d, e) Immunofluorescence staining with MyoD (red) (d) or Desmin (red) (e) and DAPI (blue) on Day 14 comparing 5-Aza induction with or without Noggin (200 ng/mL), respectively. Arrowheads indicate merged MyoD-positive myonuclei; white dotted line indicates cell outline; green dotted circle indicates Desmin-positive merged myonuclei; white scale bar, 50 µm; green scale bar, 10 µm. (f, g) Percentage of MyoD-positive cells (f) or Desmin-positive myotube-like cells (g) is presented as the ratio of nuclei to total nuclei (*n* = 4). (h) Protein expression of Desmin and MRF4 was assessed using western blot on Days 14 and 21. (i, j) Semiquantitative analysis of the expression ratio of (i) Desmin/*β*-actin and (j) MRF4/*β*-actin (*n* = 4). The differences between blank and 5-Aza or between 5-Aza, 5-Aza + 100N and 5-Aza + 200N were presented as *⁣*^*∗*^*p* < 0.05 and *⁣*^*∗∗*^*p* < 0.01. 100N = 100 ng/mL Noggin; 200N = 200 ng/mL Noggin. DPSCs, dental pulp stem cells; MyoD, myogenic differentiation 1.

To evaluate the effect of Noggin treatment on the MyoD of DPSCs, we assessed the expression of myogenic genes such as MyoD, MRF4, and Desmin on Days 7, 14, and 21. Noggin increased the expression of MyoD and Desmin (Figure 2a,b) but had no effect on the mRNA expression of MRF4 on Day 7 (Figure 2c). Immunofluorescence staining for MyoD (Figure 2d) and Desmin (Figure 2e) revealed elevated levels of MyoD (Figure 2f) and an increased quantity of long, spindle-shaped myotube-like cells (Figure 2g) in the Noggin-treated groups. Moreover, the protein expression levels of Desmin (Figure 2j) and MRF4 (Figure 2k) significantly increased upon treatment with Noggin on Day 14 (Figure 2h). In our study, Noggin was shown to play an important regulatory role in accelerating the MyoD of DPSCs, which might be a novel factor in myogenesis.

### 3.3. Noggin Increases the Generation of Pax7^+^ Satellite-Like Cells in DPSCs

SCs are characterized by the expression of the paired box (Pax)3/7. Six1 and eya2 have been shown to activate SCs (Pax7^+^) and promote myoblast differentiation. Notably, we found that Noggin increased the protein levels of Pax7 in DPSCs cultured in a proliferation medium (Figure S3). To determine SCs generation through MyoD of DPSCs with Noggin, we measured the relative mRNA levels on Days 1, 3, and 7, as well as the protein expression levels of Six1, Pax3, Pax7, and Eya2 on Days 7, 14, and 21.

**Figure 3:**
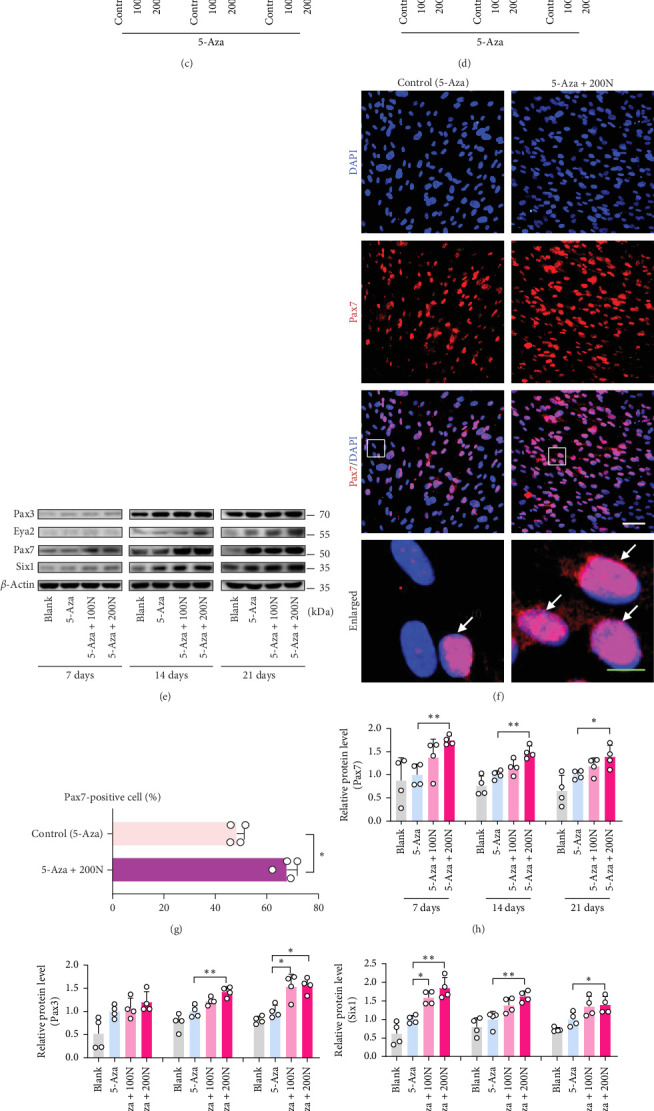
The generation of satellite-like cells in DPSCs. (a)–(d) Relative mRNAs of (a) Pax7, (b) Pax3, (c) Six1, and (d) Eya2 were assessed on Days 1, 3, and 7 using quantitative PCR. (e) Protein expression of Pax7, Pax3, Six1, and Eya2 was assessed using western blotting on Days 7, 14, and 21 (*n* = 3). (f) Immunofluorescence staining with Pax7 (red) and DAPI (blue) on Day 14 comparing 5-Aza induction with or without Noggin (200 ng/mL), respectively. Arrowheads indicate merged Pax7-positive myonuclei; white scale bar, 50 µm; green scale bar, 10 µm. (g) Percentage of nuclei is presented as the ratio of Pax7-positive nuclei to total nuclei (*n* = 4). (h–k) Semiquantitative analysis of the expression ratio of (h) Pax7/*β*-actin, (i) Pax3/*β*-actin, (j) Six1/*β*-actin, and (k) Eya2/*β*-actin (*n* = 4). The differences between blank and 5-Aza or between 5-Aza, 5-Aza + 100N and 5-Aza + 200N were presented as *⁣*^*∗*^*p* < 0.05 and *⁣*^*∗∗*^*p* < 0.01. 100N = 100 ng/mL Noggin; 200N = 200 ng/mL Noggin. DAPI, 4′,6-diamidino-2-phenylindole; DPSCs, dental pulp stem cells; PCR, polymerase chain reaction.

The mRNA expression of Pax7 increased in a concentration-dependent manner under Noggin treatment, reaching its peak on Day 3 and remaining higher than that of the control on Day 7 (Figure 3a). Consistent with the expression of Pax7, the mRNA expression of Pax3 increased on Day 3 and remained elevated on Day 7 (Figure 3b). Additionally, the mRNA expression of Six1 (Figure 3c) and Eya2 (Figure 3d) increased on Day 7. Western blotting (Figure 3e) and immunofluorescence staining (Figure 3f) for Pax7 revealed that Noggin upregulated the protein expression of Pax7 on Days 7, 14, and 21 (Figure 3g,h). The protein expression levels of Pax3 (Figure 3i), Six1 (Figure 3j), and Eya2 (Figure 3k) were also increased in the Noggin-treated groups on Day 14 and remained high on Day 21 (Figure 3e). These results suggest that Noggin promotes the myogenic process in DPSCs by upregulating the expression of Six1/Eya2 in addition to Pax3/Pax7.

### 3.4. Noggin Antagonizes BMP by Downregulating p-Smad 1/5/9, Thus Facilitating the MyoD of DPSCs

**Figure 4:**
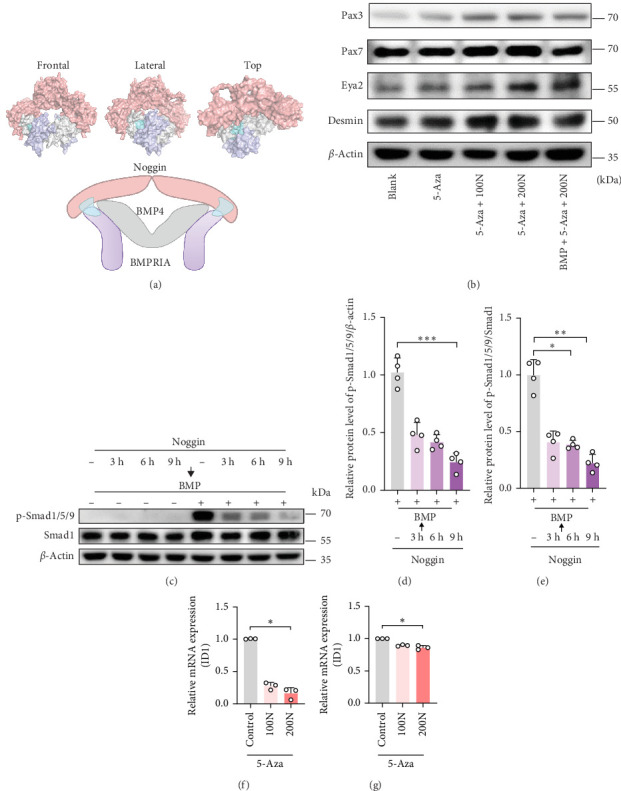
Noggin antagonize BMP's inhibition of myogenic differentiation of DPSCs. (a) 3D protein structure of Noggin's competitive inhibition of BMP4 binding to BMPRIA. Red indicates Noggin. Gray indicates BMP4. Purple indicates BMPRIA. Cyan indicates a competitive site of Noggin binding to BMPRIA. (b) Protein expression of Pax7, Pax3, Eya2, and Desmin was assessed using a western blot when rescuing with BMP4. (c–e) Protein expression of p-Smad1/5/9 and Smad1 was assessed using western blot by Noggin treatment for different times (3, 6, and 9 h) and subsequently stimulated with BMP4 or control for 1 h (c), and semiquantitative analysis of the expression ratio of (d) p-Smad1/5/9/*β*-actin, (e) p-Smad1/5/9/Smad1 were performed (*n* = 4). (f, g) Relative mRNAs expression of (f) ID1 and (g) MSX1 were assessed using quantitative PCR (*n* = 3). The differences were presented as *⁣*^*∗*^*p* < 0.05 and *⁣*^*∗∗∗*^*p* < 0.001. 100N = 100 ng/mL Noggin; 200N = 200 ng/mL Noggin. BMP, bone morphogenetic protein; BMPRIA, BMP4 to BMP-receptor I A; DPSCs, dental pulp stem cells; PCR, polymerase chain reaction.

To determine whether Noggin regulates the MyoD of DPSCs by regulating Smad signaling, we first simulated 3D protein structures using SWISS-MODEL and visualized them with PyMOL software, which showed that Noggin competitively inhibits the binding of BMP4 to BMP-receptor I A (BMPRIA) (Figure 4a). Then, we blocked the effect of Noggin by adding the BMP protein. The protein levels of Pax7, Pax3, Eya2, and Desmin were observed (Figure 4b). Compared with that of the Noggin treatment group (200N), their expression decreased in the Noggin + BMP treatment group. Among them, Pax7 was most prominent when compared with that in the 5-Aza or control groups (Figure 4b).

To further explore the downstream regulation of BMP signaling by Noggin, we determined the phosphorylation levels of members of the BMP/p-Smad pathway (Figure 4c−e). The protein levels of p-Smad 1/5/9 increased following the activation of the BMP pathway by adding BMP4 (Figure 4c). Noggin persistently eliminated the phosphorylation levels even after subsequent stimulation with BMP4 (Figure 4c−e). Downstream effectors of the BMP/p-Smad pathway, such as inhibitors of DNA binding 1 (ID1) and msh homeobox 1 (MSX1), were also downregulated by Noggin treatment (Figure 4f,g). Therefore, our results indicate that Noggin facilitates the MyoD of DPSCs by inhibiting BMP/Smad activation.

### 3.5. Noggin-Pretreated DPSCs Combined With Matrigel Can Effectively Repair Muscle Injury in VML

**Figure 5:**
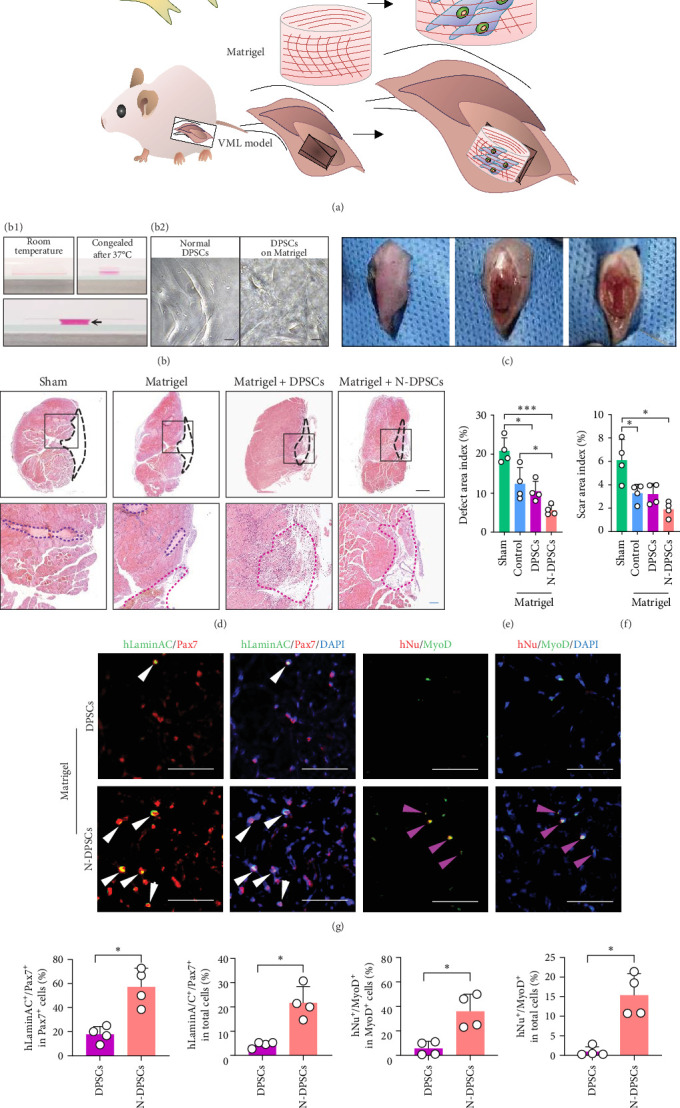
Noggin-pretreated DPSCs improved muscle repair on VML. (a) Schematic diagram of VML surgery and transplantation of a bioconstruct containing cells and Matrigel into the tibialis anterior muscle. (b) Characteristics of Matrigel at room temperature or congealed after 37°C (b1), and DPSCs cultured on common 96-well culture dish or Matrigel (b2). Arrowheads indicate space. Scar Bar, 10 μm. (c) VML surgery of tibialis anterior muscle. (d) H&E staining of tibialis anterior muscle cross-sections. Large dotted black line indicates that the area removed in the surgery but still remains defect; black box in the left column indicates the image area of the right column; small dotted purple line indicates defect collagenous scar tissue; small dotted red line indicates repairing tissue infiltrated by diverse cells; black scale bar, 500 µm; blue scale bar, 100 µm; purple scale bar, 50 µm. (e) Defect area index was presented as the ratio of defect area to total muscle area (*n* = 4). (f) Scar area index was presented as the ratio of scar area to total muscle area (*n* = 4). (g) Donor-derived Pax7^+^ satellite-like cells were detected by human-specific LaminA/C and Pax7 coimmunostaining. Donor-derived MyoD^+^ myoblasts were detected by hNu and MyoD coimmunostaining. White arrowheads indicate hLaminA/C^+^/Pax7^+^ cells. Purple arrowheads indicate hNu^+^/MyoD^+^ cells. Scale bar, 20 µm. (h–k) Quantification of hLaminA/C^+^/Pax7^+^ in Pax7^+^ cells (h) or in total cells (i), and hNu^+^/MyoD^+^ in MyoD^+^ cells (j) or in total cells (k). The differences were presented as *⁣*^*∗*^*p* < 0.05*⁣*^*⁣⁣*^ and *⁣*^*∗∗∗*^*p* < 0.001. N-DPSCs, Noggin-treated dental pulp stem cells; VML, volumetric muscle loss.

To test the utility of DPSCs in repairing muscle injuries, we established a mouse VML injury model that exhibited a loss of ~40% of the tibialis anterior muscle (Figure 5a−c). In the untreated VML-injured group, the volumetric defect remained (Figure 5d). Shrinkage occurred due to the defect area. The muscle defect deformity exhibited the deposition of a thin layer of disorganized collagenous scar tissue (Figure 5d). The injury defect exceeded the threshold and could not be restored by the endogenous regenerative potential of the skeletal muscle. This result indicates the success of VML modeling.

The utilization of scaffolds can provide temporary mechanical support and the necessary growth environment for seed cell adhesion, growth, proliferation, and differentiation. Therefore, we used the thermosensitive hydrogel Matrigel to provide a scaffold for stem cell transplantation (Figure 5d). We observed that the Matrigel group exhibited a reduced shrinkage area and decreased muscle fibrosis (Figure 5d). In addition, we found some mononuclear cells infiltrating in the defect (Figure 5d).

To examine whether Noggin-pretreated DPSCs could better repair muscle injury than the controls, we transplanted DPSC bioconstructs into the defects of VML muscles (Figure 5a–c). We implanted untreated DPSCs (Figure 5d) or Noggin-pretreated DPSCs (Figure 5d) reconstituted in Matrigel into the defects. Morphometric analysis of muscle cross-sections revealed that the Noggin-treated DPSCs showed a 73% decrease in the size of the defect and a 69% decrease in scar tissue (Figure 5d–f). In contrast to the defects in the above two groups, which exhibited irreversible and robust fibrotic scars, the defects treated with DPSCs showed markedly reduced fibrotic tissue surrounded by cells of varying morphologies, such as fibrotic, inflammatory, and vessel-like cells, indicating the tissue repair process (Figure 5d). In contrast, Noggin-treated DPSCs might have accelerated this process and facilitated the improved formation of muscle tissue, leaving little cell infiltration (Figure 5d).

### 3.6. Noggin-Pretreated DPSCs Bioconstructs Can Benefit the Muscle SC Population and Promote Myogenic Repair

To explore the contribution of grafted cells to muscle injury, we immunostained muscle cross-sections for Pax7 (SC marker), MyoD (activated SCs/myoblasts), hNu, and human LaminA/C (specific antibody to trace human cells), and Laminin (to identify position within the sarcolemma). Pax7/MyoD costaining revealed that stem cell transplantation increased the proportion of activated SCs compared to the sham or Matrigel groups (Figure S4). We also observed that hNu was integrated into the nucleus of regenerated tissue and was located on the Laminin-stained muscle sarcolemma (Figure S4), a phenomenon more readily discernible in the Noggin-treated DPSCs groups than in the DPSCs groups. Increased numbers of hLaminAC^+^/Pax7^+^ (Figure 5g) and hNu^+^/MyoD^+^ (Figure 5g) cells were also found in the Noggin-treated DPSCs groups compared with the DPSCs groups, indicating an increase in donor-derived SCs (Figure 5h−k). These results suggest that Noggin-treated DPSCs partially benefited the SC population. This benefit might provide support for muscle regeneration in VML injury.

## 4. Discussion

In our study, we found that Noggin could promote the MyoD of DPSCs and increase the generation of satellite-like cells by regulating the BMP/Smad signaling pathway. Then, we treated VML model mice with Noggin-pretreated DPSCs bioconstructs and found that Noggin pretreatment improved the repair of muscle injury. Our work implies that Noggin can promote the MyoD of DPSCs, rendering DPSCs an important source of muscle stem cells for muscle repair.

Noggin, a secreted BMP antagonist, was promoted by Wnt-1 in medial somites and found to promote the expression of MyoD in embryonic tissues [21, 23]. Our results showed that Noggin could promote the MyoD of DPSCs (Figures 1 and 2) and increase the number of Pax3^+^/Pax7^+^ cells (Figure 3). Previous studies have concluded that Pax3-mediated myogenesis requires an environment where Six1 synergizes with Eya2 to activate the expression of MyoD [28]. Our results showed that Noggin promoted the expression of Six1 and Eya2, suggesting that these Pax3^+^/Pax7^+^ cells were in an environment conducive to subsequent MyoD. We also found that Noggin eliminated Smad phosphorylation, accompanied by decreased levels of MSX1 and ID1 (Figure 4). Consistent with our results, Noggin inhibited p-Smad1/5/8 and repressed ID1 and MSX1 expression, impeding myoblast differentiation [29–31]. Sustained release of BMP4 significantly decreased Pax7^+^ and MyoD^+^ SC densities [32]. When BMP4 was added to antagonize Noggin, we observed inhibition of the MyoD of DPSCs. Our data revealed that Pax3/7 might be related to the BMP/Smad pathway in the context of Noggin effects. Previous research has shown that activated Pax7^+^ cells coexpressed p-Smad1/5/8 in injured mouse skeletal muscle sections [31]. In the competition for MSX1, the expression of downstream effectors of the BMP/Smad pathway, Pax3 or Pax7, was increased towards the myogenic fate [33]. Knockout of Noggin leads to increased levels of p-Smad1/5/8, ID1, and MSX1, and a decreased population of mesenchymal Pax7^+^ muscle precursor cells [25].

**Figure 6:**
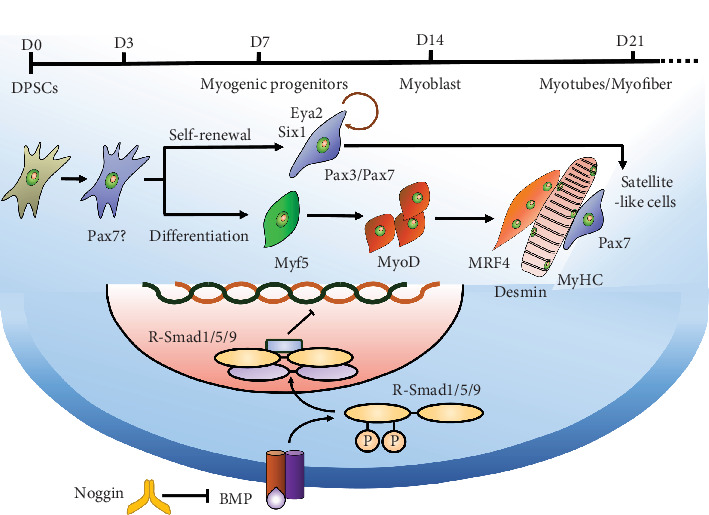
The possible mechanism of Noggin regulating myogenic differentiation of DPSCs. Noggin regulates the phosphorylation of Smad 1/5/9, so as to promote the myogenic differentiation of DPSCs. This way of differentiation preserves the maintenance of Pax7^+^ satellite-like cells in DPSCs. Diagram mapping the developmental stages of myogenic differentiation from DPSCs, accompanied by a timeline of myogenic markers. DPSCs, dental pulp stem cells.

Muscle regeneration is ensured by Pax7^+^ SCs; when activated, SCs are able to divide asymmetrically, enabling self-renewal or myoblast differentiation [1]. ESCs-derived Pax7^+^ cells can give rise to both muscle fibers and Pax7^+^ satellite-like cells when grafted in vivo, suggesting that these cells might behave like SCs [34]. In our study, we found that Noggin expanded Pax7^+^ cells in DPSCs. After 21 days of differentiation in vitro, the expression levels of late myogenic marker genes (MyHC) and SC marker (Pax7) were both higher with Noggin treatment (Figures 1 and 3). These results revealed that Noggin could promote the generation of Pax7^+^ satellite-like cells from DPSCs (Figure 6).

We then transplanted Noggin-pretreated DPSCs combined with Matrigel into defects of a VML injury model. In our study, Noggin-pretreated DPSCs bioconstructs decreased the size of the defect and increased the number of donor-derived hLaminAC^+^/Pax7^+^ and hNu^+^/MyoD^+^ cells in mouse muscles (Figure 5g). Some scholars transplanted ESC- or PSC-derived muscle progenitor cells into NSG-mdx4cv mice and found that donor-derived hLaminAC^+^/Pax7^+^ SCs contributed to the muscle stem cell pool in tissues [35]. The successful use of Pax7^+^ satellite-like cells derived from ESCs in muscle injury may be attributed to their self-renewal capacity within the SCs pool [36].

DPSCs are a type of MSCs from dental pulp, with easy access, long-term storage, and high proliferative and differentiation potential compared to other types of MSCs [9–12] and can serve as a proper stem cell source for muscle regeneration. SCs derived from MSCs were shown to give rise to differentiated muscle fibers [37]. However, the contribution of 5-Aza-induced DPSCs engraftment might only be a paracrine effect instead of MyoD [14]. In contrast to muscle-derived cells or ESCs, the transplant efficiency of MSCs was relatively low, with ~1%–2% as hybrid myofibers [38]. We speculate that the low myogenic efficiency may be attributed to fewer SCs. Our results indicated that Noggin expanded Pax7^+^ satellite-like cells in DPSCs, which increased the percentage of donor-derived cells to ~15%–20% (Figure 5i–k). This finding suggests that Noggin-treated DPSCs can partially benefit the muscle SC pool and promote myogenic repair in VML injury.

## 5. Conclusions

In this study, we demonstrate that Noggin, a secreted BMP antagonist, is an important protein that promotes MyoD and increases the generation of Pax7^+^ satellite-like cells by modulating BMP/Smad signaling. These satellite-like cells combined with Matrigel can effectively repair mouse tibialis anterior muscle injury in a VML model, leading to increased repair size and decreased scar tissue. Noggin-treated DPSCs bioconstructs can benefit the Pax7^+^ SC pool and promote muscle regeneration. This finding suggests the importance of producing Pax7^+^ SCs and highlights the potential of these SCs for improved muscle regeneration. Noggin might enhance the generation of satellite-like cells from DPSCs to provide potential transplantable stem cells for muscle regeneration. Further investigations are needed to study the relationship between Pax7 and Smad/its downstreams in order to improve myogenic induction conditions and enhance the transplantation of DPSCs into damaged muscle tissue.

## Data Availability

All supporting data are included in the article and its additional files.

## Supporting Information

Supporting Information Figure S1: Isolation, culture, and identification of DPSCs were described. Figure S2: The mRNA and protein expression of Myf5 and Desmin were assessed after 5-Aza induction. It is proved that 5-Aza can induce myogenic differentiation of DPSCs. Figure S3: Cell cycle distribution was monitored using flow cytometry after Noggin treatment. Noggin has no effect on the cell proliferation of DPSCs. Figure S4: Immunostaining of Pax7/MyoD and hNu/Laminin in tibialis anterior muscle cross-sections were performed. Grafted cells were identified by hNu, and Noggin-pretreated DPSCs can benefit Pax7^+^/MyoD^+^ cells on mice VML. Table S1: Donor information. Table S2: The primer sequences for PCR amplification.
