# Supplementary material for: Noggin Combined With Human Dental Pulp Stem Cells to Promote Skeletal Muscle Regeneration
Source: Stem Cells Int. 2024 Dec 28;2024:2812390. doi: 10.1155/sci/2812390 (PMC11699990; doi:10.1155/sci/2812390)
Supplement: Supporting Information — Figure S1: Isolation, culture, and identification of DPSCs were described. Figure S2: The mRNA and protein expression of Myf5 and Desmin were assessed after 5-Aza induction. It is proved that 5-Aza can induce myogenic differentiation of DPSCs. Figure S3: Cell cycle distribution was monitored using flow cytometry after Noggin treatment. Noggin has no effect on the cell proliferation of DPSCs. Figure S4: Immunostaining of Pax7/MyoD and hNu/Laminin in tibialis anterior muscle cross-sections were performed. Grafted cells were identified by hNu, and Noggin-pretreated DPSCs can benefit Pax7+/MyoD+ cells on mice VML. Table S1: Donor information. Table S2: The primer sequences for PCR amplification. [file 2812390.f1.docx]

**Supporting Information**

This file contains Supplementary Sections including **Figure S1-S4** and **Table S1-2**. Isolation, culture and identification of DPSCs was described (**Figure S1**). It is proved that 5-Aza can induce myogenic differentiation of DPSCs (**Figure S2**), and Noggin has no effect on cell proliferation of DPSCs (**Figure S3**). Noggin-pretreated DPSCs can benefit Pax7^+^/MyoD^+^ cells on mice VML (**Figure S4**). Donor information (**Table S1**) and the primer sequences for PCR amplification (**Table S2**) were also listed.


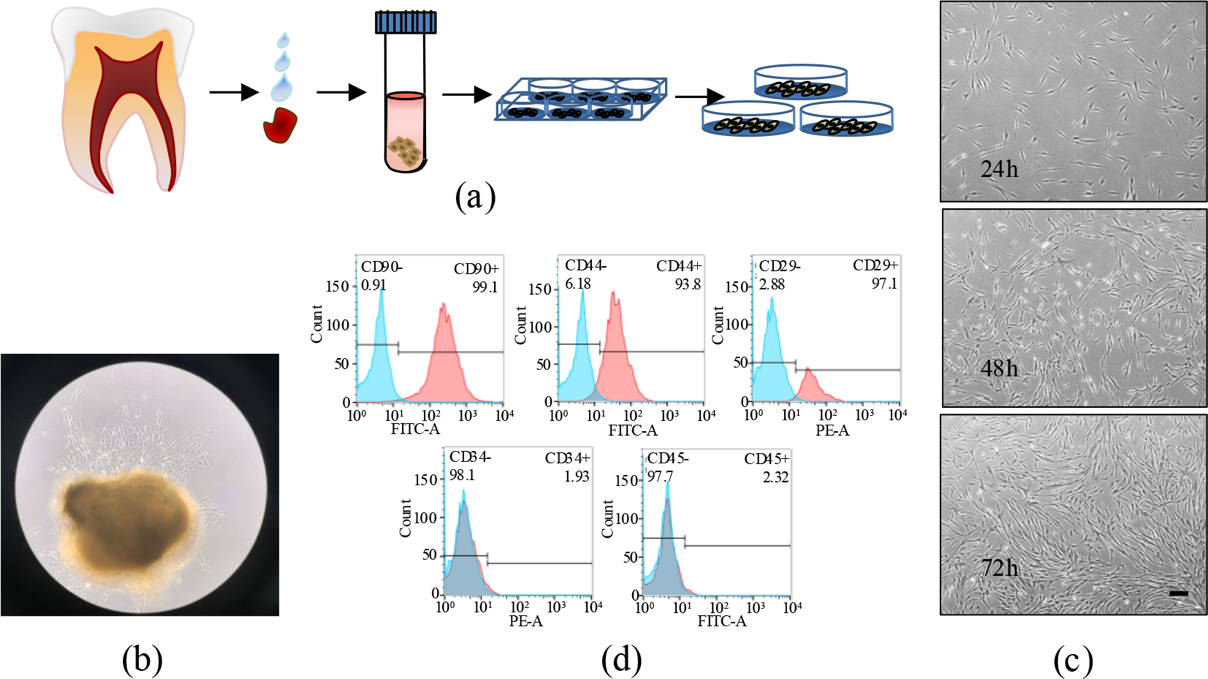


**Supporting Information Figure S1** Isolation, culture and identification of human DPSCs. (a) Schematic diagram of DPSCs extraction from dental pulp tissue and isolation by collagenase digestion. (b) Microscopic picture of DPSCs from dental pulp tissue. (c) Cell morphology of DPSCs cultured 24 h, 48 h and 72 h. Bar, 50 μm. (d) Flow cytometric analysis of the surface markers of DPSCs: positive for CD29, CD44, CD90 and negative for CD34, CD45.


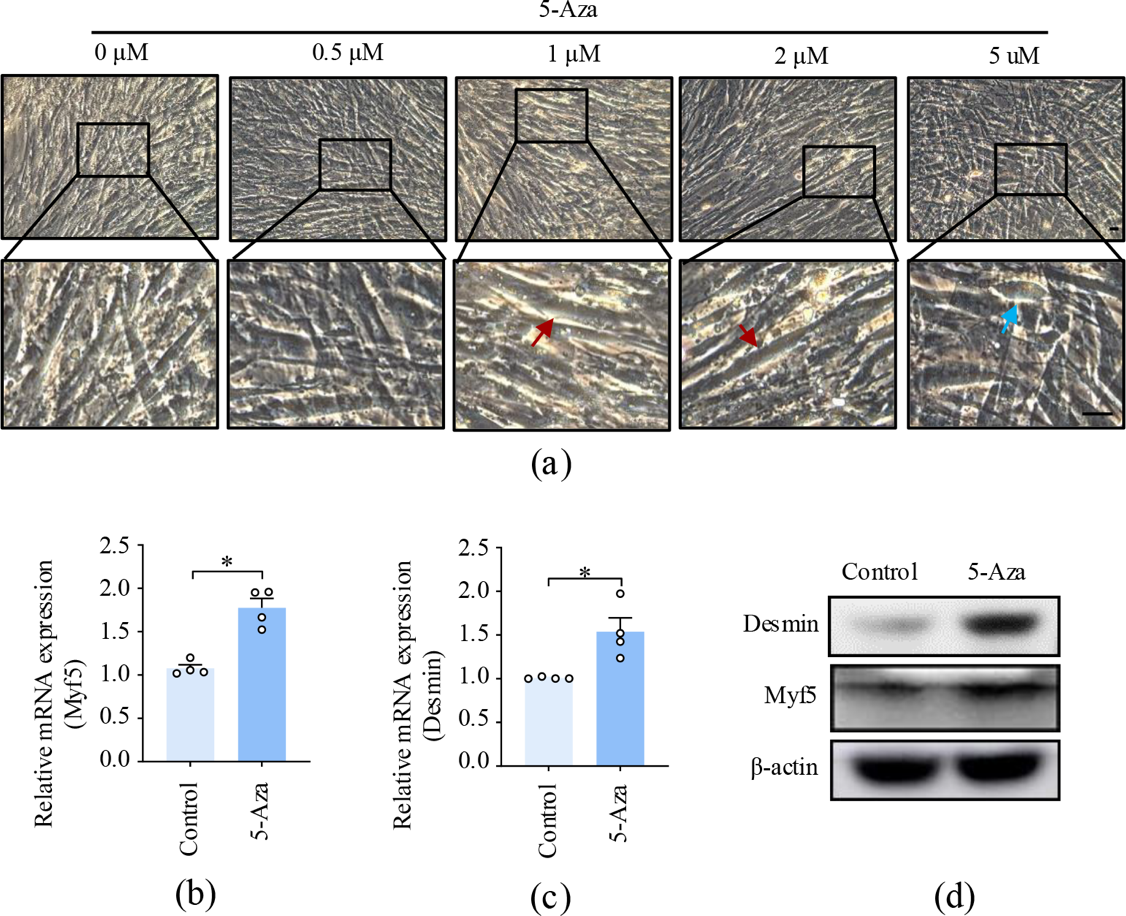


**Supporting Information Figure S2** 5-Aza induces myogenic differentiation of human DPSCs. (a) Cell morphology of DPSCs induced by different concentrations of 5-Aza for 21 day-myogenic differentiation. Red arrows indicated myotube-like cells; Blue arrows indicated atrophy formed-myotube; Scale bar, 20 µm. (b)-(c) The mRNA expression of Myf5 (b) and Desmin (c) were assessed with or without 1 μM 5-Aza induction using quantitative PCR. Data are presented as the fold‑change of the control group without 5-Aza treatment (n = 4). (d) Protein expression of Desmin (day 21) and Myf5 (day 14) was assessed using western blotting with or without 1 μM 5-Aza induction. *P < 0.05, ***P* < 0.01, and ****P* < 0.001

**
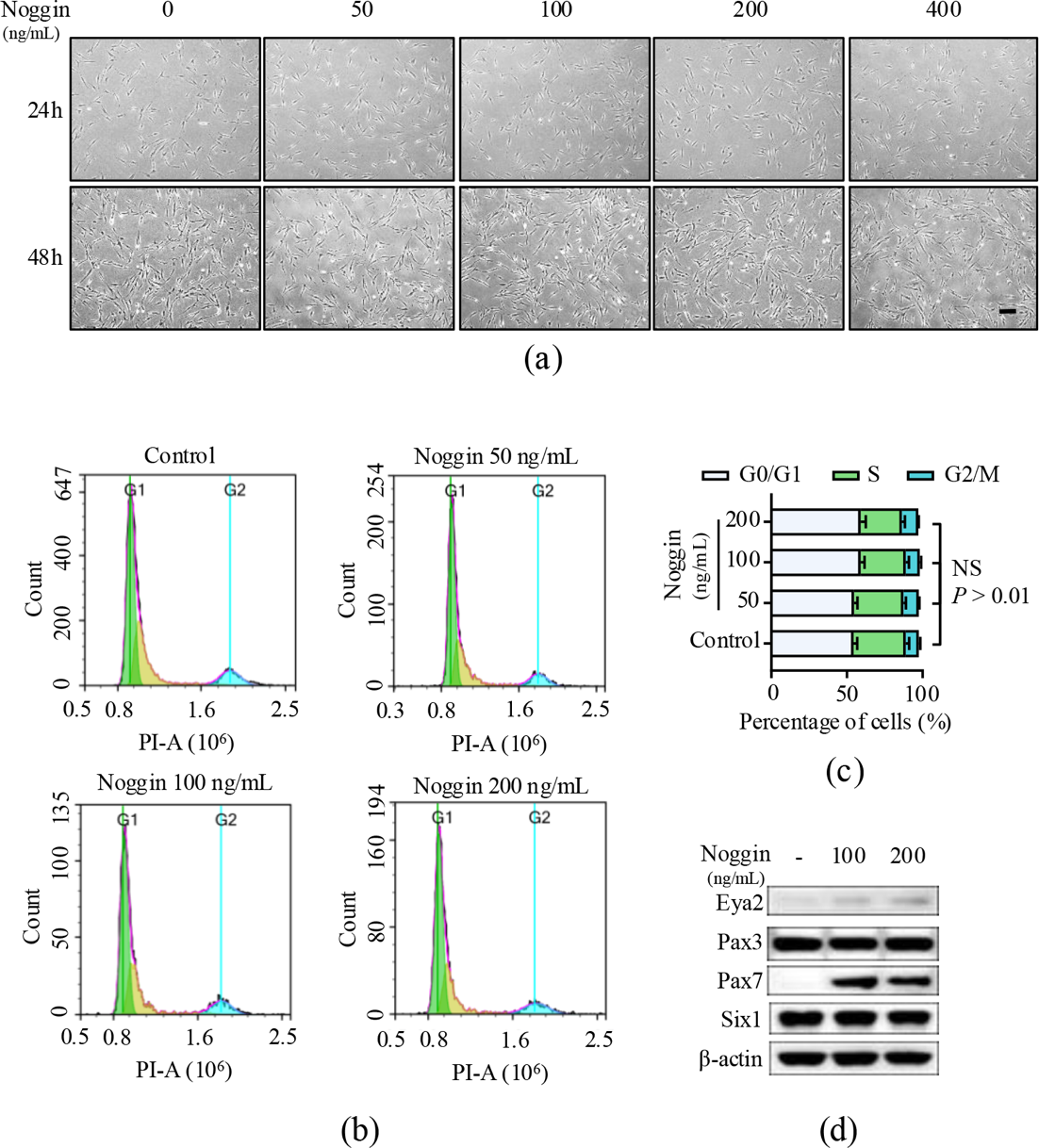
**

**Supporting Information Figure S3** Noggin has no effect on cell proliferation of DPSCs. (a) Cell morphology of DPSCs cultured 24h and 48h with different concentrations of Noggin (50 ng/mL, 100 ng/mL, 200 ng/mL or 400 ng/mL). Scar bar, 100 μm. (b) Cell cycle distribution was monitored using flow cytometry (n = 3). (c) Statistical analysis of the cell cycle (*n* = 3). (d) Protein expression of Eya2, Pax7, Pax3, and Six1 was assessed using western blotting in DPSCs under growth medium with different concentration of Noggin.


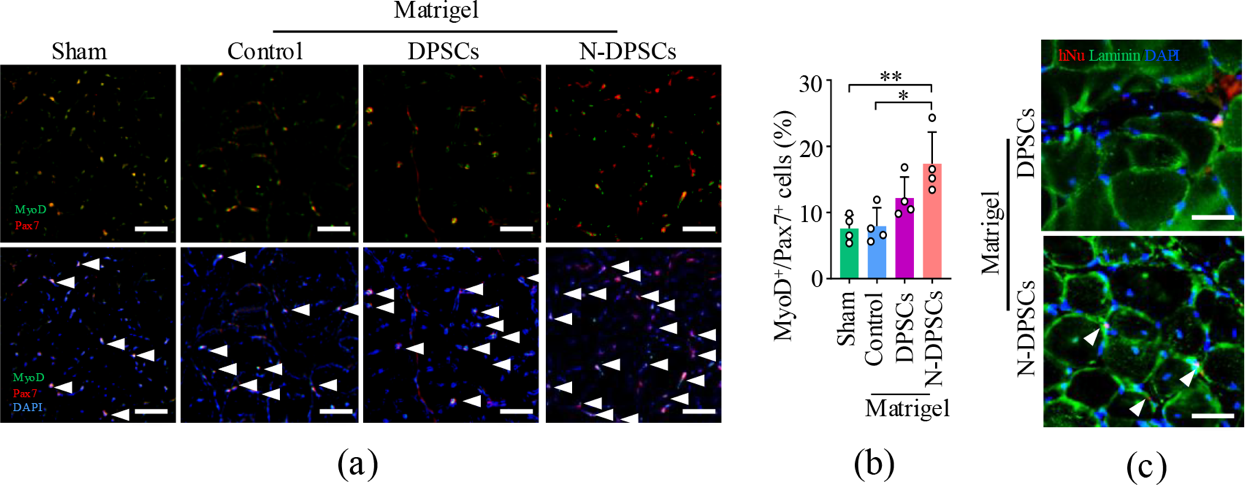


**Supporting Information Figure S4** Noggin-pretreated DPSCs can benefit Pax7^+^/MyoD^+^ cells on mice VML. (a) Representative Pax7 and MyoD immunostaining of tibialis anterior muscle cross-sections (n = 4). (b) Quantification of Pax7^+^/MyoD^+^ cells. White arrowheads indicate Pax7^+^/MyoD^+^ cells. Scale bar, 20 µm. (c) Grafted cells were identified by human Nucleoli (hNu) and by co-expression of hNu and Laminin. Arrowheads indicate hNu^+^ nuclear. Scale bar, 20 µm. The differences were presented as **P* < 0.05, ***P* < 0.01 and ****P* < 0.001. VML = volumetric muscle loss; N-DPSCs = Noggin-treated DPSCs.

The donors sought for dental treatment in Shanghai Stomatological Hospital were recruited. The extracted teeth were those needed be removed for orthodontic treatment, or the wisdom teeth for dental preventive care. It has been approved by Ethics Committee (No. 2017-0004 and No. 2019-003). We have obtained informed consents to participate from the donors. The age of the patients ranged from 18 to 25 years old (see Table below). Collected teeth were free of caries or periodontal diseases.

**Supporting Information Table S1.** Donor information

| Age range/years | Gender | Recruitment date | Tooth position |
| --- | --- | --- | --- |
| 18-20 | 1 | 30/04/2018 | Right upper third molar |
| 18-20 | 2 | 28/04/2018 | Right upper third molar |
| 24-25 | 2 | 28/04/2018 | Right upper third molar |
| 24-25 | 2 | 28/04/2018 | Right lower third molar |
| 24-25 | 2 | 21/04/2018 | Right lower third molar |
| 21-23 | 1 | 18/09/2019 | Left upper third molar |
| 21-23 | 1 | 18/09/2019 | Left upper third molar |
| 18-20 | 2 | 09/11/2019 | Right upper first premolar |
| 18-20 | 2 | 09/11/2019 | Left upper third molar |
| 21-23 | 1 | 17/11/2019 | Right upper first premolar |
| 24-25 | 2 | 17/11/2019 | Right upper first premolar |

**Supporting Information Table S2.** Sequence of primers used for PCR amplification

| Gene | Forward (5'‑3') | Reverse (5'‑3') |
| --- | --- | --- |
| Desmin | GACCATCGCGGCTAAGAAC | GTGTAGGACTGGATCTGGTG |
| MRF4 | GGAGCGCCATCAGCTATATTG | ATCCGCACCCTCAAGATTTTC |
| MyoD | GCAACGCCATCCGCTATATC | TGTAGTCCATCATGCCGTCG |
| Pax7 | ACCCCTGCCTAACCACATC | AGCGGCAAAGAATCTTGGAG |
| Pax3 | AGCTCGGCGGTGTTTTTATCA | CTGCACAGGATCTTGGAGAC |
| Six1 | CTGCCGTCGTTTGGCTTTAC | GCTCTCGTTCTTGTGCAGG |
| Eya2 | AGCGATTGTCTGGATAAACTG | AGGTGGGTAAGCTGTATAGG |
| ID1 | CTGCTCTACGACATGAACGG | GAAGGTCCCTGATGTAGTCG |
| MSX1 | TCAAGCTGCCAGAAGATGCG | GGCTTACGGTTCGTCTTGTG |
| MYH4 | CTTGAAGTAGTTGTCTGCTTTGAGC | TTTCGGAGGAAAGGAGCAGC |
| Myf5 | AACCCTCAAGAGGTGTACCAC | GGACTGTTACATTCGGGCATG |
| β-actin | CCAACCGCGAGAAGATGA | CCAGAGGCGTACAGGGATAG |
